# Complete Genome Sequence of Bacillus velezensis Strain AL7, a Biocontrol Agent for Suppression of Cotton *Verticillium* Wilt

**DOI:** 10.1128/MRA.01595-19

**Published:** 2020-02-20

**Authors:** Haiyang Liu, Qingchao Zeng, Wei Wang, Renfu Zhang, Ju Yao

**Affiliations:** a Institute of Plant Protection, Xinjiang Academy of Agricultural Sciences, Urumqi, China; b Beijing Advanced Innovation Center for Tree Breeding by Molecular Design, Beijing Forestry University, Beijing, China; University of Rochester School of Medicine and Dentistry

## Abstract

Bacillus velezensis AL7, isolated from cotton soil, had strong antagonistic activity to *Verticillium dahlia* Kleb. The AL7 genome consisted of one chromosome with 3,894,709 bp (46.64% G+C content). Genome annotation predicted 3,706 protein-coding genes, 86 tRNAs, and 27 rRNAs. We sequenced and annotated the complete AL7 genome to help us better understand use of this strain.

## ANNOUNCEMENT

Vascular wilts caused by members of the genus Verticillium are among the most devastating fungal diseases worldwide. The genus *Verticillium* consists of a relatively small group of soilborne fungi, and several of them cause wilt disease in a variety of plant hosts, including tomato, potato, and cotton (1). *Verticillium* wilt, a devastating disease of cotton caused by the soilborne fungal pathogen Verticillium dahliae Kleb, is a serious disease that is responsible for severe economic losses in cotton production areas throughout the world (2). Control of *Verticillium* disease is difficult due to the long persistence of the resting structures in the field and the broad host ranges of some species (1). The management of this disease includes the use of resistant cultivars, the application of chemical fungicides, and crop rotation (3). Unfortunately, these methods have their specific concerns and limitations. Although several fungicides have been reported to be effective against *Verticillium* diseases, it is not easy to control *Verticillium* diseases because fungicides are not able to affect the pathogen once it enters the xylem (4). Moreover, extensive use of fungicides in agriculture systems has raised public concerns over the environment and human health. Therefore, there is a need for new environmentally friendly technologies and products to partly or fully replace chemical-based pesticides and contribute to safer crop disease control (5). Many papers have revealed that a number of biocontrol strains, including strains of Pseudomonas, Bacillus, and Streptomyces, were able to significantly reduce *Verticillium* disease (3, 6, 7). Biological preparations from spore-forming *Bacillus* spp. are preferred because their long-term viability facilitates the development of commercial products (8). In this study, we report the complete genome sequence of Bacillus velezensis AL7, an antagonist against *Verticillium* disease, isolated from cotton soil in the city of Xinjiang, China.

A single colony was incubated and cultured overnight in Luria-Bertani broth at 37°C prior to extraction. Genomic DNA of B. velezensis AL7 was extracted using the phenol-chloroform method (9). The quantity of DNA was measured with a NanoDrop 2000 instrument. Then, the DNA was sent to Beijing Novogene Bioinformatics Technology Co., Ltd., and sequenced using PacBio Sequel and Illumina HiSeq 4000 systems. Library preparation was performed with a SMRTbell template kit 1.0 (Pacific Biosciences), followed by single-molecule real-time (SMRT) sequencing on the Sequel platform. Additional Illumina sequence data were generated with a HiSeq (Illumina) 350-bp paired-end run using whole-genome shotgun libraries constructed with a NEBNext Ultra DNA library prep kit following the manufacturer’s recommendations. SMRTlink v5.0 (minLength, 50; minReadScore, 0.8) and readfq v10 (default parameters) software were used to filter the low-quality reads (10, 11). After quality filtering, the Illumina sequencing generated a total of 4,033,206 paired reads (150 bp). Meanwhile, the single-molecule sequencing produced 191,057 subreads, with a mean read length of 8,791 bp and an *N*_50_ length of 9,638 bp. The high-quality reads were assembled using SPAdes software and a hybrid approach combining Illumina and PacBio reads (12). For the assembly software, the k-mer was set as 127. Then, the scaffolds shorter than 300 bp were removed. Finally, we got one scaffold for the strain AL7 genome sequence. Annotation was performed using Prokka software (v1.11) using default parameters (13). Putative proteins were searched against the Clusters of Orthologous Groups (COG), NCBI non-redundant (nr) protein, Gene Ontology (GO), and Kyoto Encyclopedia of Genes and Genomes (KEGG) databases.

The complete genome sequence of strain AL7 revealed a genome size of 3,894,709 bp and a G+C content of 46.64%. The genome sequences contained 3,706 coding sequences (CDSs), 27 rRNAs, and 86 tRNAs. The predicted protein coding genes represented 88.50% of the genome sequence and had a total length of 3,446,697 bp. A total of 2,646 protein-coding genes were assigned putative functions, and 2,872 genes were categorized into COG functional groups. The average nucleotide identity was calculated using online OAT tools (https://www.ezbiocloud.net/tools/orthoani). The genome of strain AL7 was found to be closely related to that of *B. velezensis* FZB42, with an average nucleotide identity of 98.39%. *B. velezensis* AL7 can also synthesize antifungal antibiotics, and gene clusters for antibiotic synthesis were found. AntiSMASH online prediction software (https://antismash.secondarymetabolites.org/) using default parameters was used to show that several genes, including surfactin, iturin, and fengycin, were represented in the genome sequence of *B. velezensis* AL7.

The genome sequence and annotation of *B. velezensis* AL7 contributed to revealing the molecular mechanism of its antimicrobial activity, which suggests that *B. velezensis* AL7 could be used as a biocontrol agent of various plant diseases.

### Data availability.

The genome sequence of B. velezensis AL7 obtained in this whole-genome shotgun project has been deposited at NCBI under the accession number CP045926 (assembly number GCA_009663035). The version described in this paper is the first version. The raw sequencing data have been deposited in the same database under the accession numbers SRR10420793 and SRR10420792.
